# Spatio-temporal effects of estimated pollutants released from an industrial estate on the occurrence of respiratory disease in Maptaphut Municipality, Thailand

**DOI:** 10.1186/1476-072X-5-48

**Published:** 2006-11-08

**Authors:** Somchai Jadsri, Pratap Singhasivanon, Jaranit Kaewkungwal, Rattana Sithiprasasna, Somkiat Siriruttanapruk, Supawadee Konchom

**Affiliations:** 1 Department of Tropical Hygiene, Faculty of Tropical Medicine, Mahidol University, Rajvithi Road, Bangkok, Thailand; 2 Mosquito Biology Section, Department of Entomology, Armed Forces Research Institute of Medical Science (AFRIMS), Bangkok, Thailand; 3 Research and Development Section, Bureau of Occupational and Environmental Disease, Department of Disease Control, Ministry of Public Health, Bangkok, Thailand; 4 Malaria Division, Department of Disease Control, Ministry of Public Health, Bangkok, Thailand

## Abstract

**Background:**

Maptaphut Industrial Estate (MIE) was established with a single factory in 1988, increasing to 50 by 1998. This development has resulted in undesirable impacts on the environment and the health of the people in the surrounding areas, evidenced by frequent complaints of bad odours making the people living there ill. In 1999, the Bureau of Environmental Health, Department of Health, Ministry of Public Health, conducted a study of the health status of people in Rayong Province and found a marked increase in respiratory diseases over the period 1993–1996, higher than the overall prevalence of such diseases in Thailand. However, the relationship between the pollutants and the respiratory diseases of the people in the surrounding area has still not been quantified. Therefore, this study aimed to determine the spatial distribution of respiratory disease, to estimate pollutants released from the industrial estates, and to quantify the relationship between estimated pollutants and respiratory disease in the Maptaphut Municipality.

**Results:**

Disease mapping showed a much higher risk of respiratory disease in communities adjacent to the Maptaphut Industrial Estate. Disease occurrence formed significant clusters centred on communities near the estate, relative to the weighted mean centre of chimney stacks. Analysis of the rates of respiratory disease in the communities, categorized by different concentrations of estimated pollutants, found a dose-response effect. Spatial regression analysis found that the distance between community and health providers decreased the rate of respiratory disease (p < 0.05). However, after taking into account distance, total pollutant (p < 0.05), SO_2 _(p < 0.05) and NO_x _(p < 0.05) played a role in adverse health effects during the summer. Total pollutant (p < 0.05) and NO_x _(p < 0.05) played a role in adverse health effects during the rainy season after taking into account distance, but during winter there was no observed relationship between pollutants and rates of respiratory disease after taking into account distance. A 12-month time-series analysis of six communities selected from the disease clusters and the areas impacted most by pollutant dispersion, found significant effects for SO_2 _(p < 0.05), NO_x _(p < 0.05), and TSP (p < 0.05) after taking into account rainfall.

**Conclusion:**

This study employed disease mapping to present the spatial distribution of disease. Excessive risk of respiratory disease, and disease clusters, were found among communities near Maptaphut Industrial Estate. Study of the relationship between estimated pollutants and the occurrence of respiratory disease found significant relationships between estimated SO_2_, NO_x_, and TSP, and the rate of respiratory disease.

## Background

Maptaphut Industrial Estate (MIE) was established by the Eastern Sea Board development project as the local area for petrochemical industries for the economic development of the country. The MIE started with a single factory in 1988, increasing to 50 by 1998.

The main air pollutants released from industries in the estate are sulfur dioxide, nitrogen oxides and total suspended particulates. These pollutants can increase respiratory symptoms and impact on the environment [1-3]. In the Maptaphut area, the pollutants negatively affect the health of people residing downwind in the area. This impact on their health was evidenced by frequent complaints of bad odour, which make people living there ill.

In 1994, Walter *et. al*. conducted a hospital-based study in Birmingham and found an association between smoke and sulfur dioxide with hospital admissions for respiratory disease [4]. One year later, they conducted another hospital-based study and found that nitrogen dioxide was significantly associated with hospital admission rates for all respiratory disease in children [5]. In addition Kramer *et. al*. [6] and Heinrich *et. al*. [7] conducted a community based study in Germany and found a similar result that respiratory disease was associated with sulfur dioxide or total suspended particulate.

**Figure 1:**
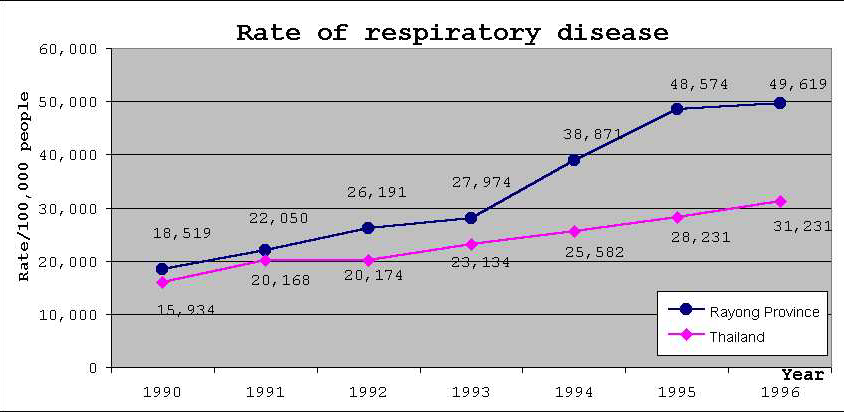
**Rate of respiratory diseases in Thailand compared to Rayong province**. Blue line indicates rates of respiratory disease of Rayong province. Magenta line indicates rates of respiratory disease of Thailand.

In 1999, the Bureau of Environmental Health, Department of Health, Ministry of Public Health, conducted a study of the health status of the people in Rayong Province, by reviewing Report 504 (outpatient disease occurrence) from Rayong Provincial Public Health Office. This temporal study found a marked increase in respiratory system diseases over the years 1993–1996, which was higher in Rayong Province than the overall occurrence of such diseases in Thailand (see figure 1).

However, it is doubtful that the findings from the Bureau of Environmental Health study and the complaints from the people residing in the area indicate conclusively that the estate was responsible. This study, conducted in Maptaphut Municipality, aimed to determine the spatial distribution of respiratory disease and pollutant dispersion across 25 communities, and the relationship between estimated pollutants and occurrence of respiratory disease.

## Results

### Smoothed relative risk in the communities

Table 1 shows that, the summer rate of respiratory disease in the study area was 56.49 per 1,000 people. The top three highest summer relative risks (RR) and rates were at Takuanaupradoo (RR = 3.38, rate = 190.72/1,000), Mapchalood (RR = 3.16, rate = 178.29/1,000) and Soiraumpattana (RR = 2.28, rate = 128.86/1,000).

**Table 1:** Rate and relative risk during summer, rainy season and winter.

Community	population	Summer			Rainy season			Winter		
		
		cases	rate	relative risk	cases	rate	relative risk	cases	rate	relative risk
Mapka	1148	26	22.72	0.40	29	25.56	0.36	42	36.62	0.48
Hauypongnai	3127	40	13.17	0.23	89	28.73	0.40	114	36.65	0.48
Nongwaisom	1235	9	8.23	0.15	16	13.95	0.19	14	12.35	0.16
Baanbon	1215	91	74.00	1.31	103	83.70	1.17	102	83.95	1.10
Saklookya	1542	58	37.72	0.67	122	79.31	1.11	135	87.81	1.15
Taladhauypong	795	97	120.29	2.13	153	190.57	2.66	157	196.10	2.58
Mapya	1080	27	25.49	0.45	44	41.35	0.58	39	36.61	0.48
Baanplong	632	48	75.43	1.34	54	84.62	1.18	52	81.90	1.08
Mapchalood	1050	188	178.29	3.16	195	185.24	2.59	238	226.57	2.98
Baanlang	1025	7	7.79	0.14	21	20.55	0.29	24	23.59	0.31
Watmaptaphut	1191	38	31.96	0.57	35	29.92	0.42	31	26.62	0.35
Samnukkabak	320	2	7.73	0.14	3	10.43	0.15	4	12.24	0.16
Watsopol	1088	76	69.77	1.24	62	57.67	0.81	71	66.00	0.87
Islam	875	35	39.78	0.70	66	75.50	1.05	109	124.00	1.63
Kaopai	830	12	14.47	0.26	14	16.63	0.23	4	5.12	0.07
Kodehin	1128	10	9.65	0.17	10	10.08	0.14	9	8.76	0.12
Taladmaptaphut	1191	56	46.69	0.83	60	50.24	0.70	29	25.05	0.33
Soiraumpattana	1088	141	128.86	2.28	155	141.27	1.97	160	146.60	1.93
Nongnamyen	537	1	5.63	0.10	1	6.70	0.09	1	5.80	0.08
Klongnamhoo	476	39	79.41	1.41	42	84.52	1.18	34	68.09	0.89
Nongbaudang	670	1	3.65	0.06	1	3.71	0.05	0	2.25	0.03
Nongpab	825	67	82.17	1.46	108	131.76	1.84	133	160.97	2.12
Takuanaupradoo	1670	319	190.72	3.38	396	236.65	3.31	381	227.96	3.00
Koakoknongtungmae	473	44	91.01	1.61	31	63.36	0.88	56	116.30	1.53
Krokyaicha	457	18	40.02	0.71	28	61.86	0.86	15	34.44	0.45

The rainy-season rate of respiratory disease, which was higher than summer, was 71.61 per 1,000 people. The top three highest rainy-season risks and rates were at Takuanaupradoo (RR = 3.31, rate = 236.65/1,000), Taladhauypong (RR = 2.66, rate = 190.57/1,000) and Mapchalood (RR = 2.59, rate = 185.24/1,000).

The winter rate of respiratory disease, the highest of the three seasons, was 76.13 per 1,000 people. The top three highest winter risks and rates were at Takuanaupradoo (RR = 3.00, rate = 227.96/1,000), Mapchalood (RR = 2.98, rate = 226.57/1,000) and Taladhauypong (RR = 2.58, rate = 196.10/1,000).

### Disease mapping and disease clustering

In order to present the magnitude of risk in the communities, the relative risk was arbitrarily grouped into four classes. Relative risk > 2 was considered highest, relative risk 1.5–2.0 was higher, relative risk 1.0–1.5 and < 1.0 were mild and no risk, respectively.

During the summer, the relative risk was > 2 in Takuanaupradoo (3.38), Mapchalood (3.16), Soiraumpattana (2.28), and Taladhauypong (2.23). Relative risk was 1.5–2 in Koakoknongtungmae (1.61). Relative risks were between 1.0–1.5 in Nongpab (1.46), Klongnamhoo (1.41), Baanplong (1.34), Baanbon (1.31) and Watsopol (1.24). In the other communities, the smoothed relative risk was < 1.0.

Spatial local clustering found three significant disease clusters. The 1^st^, indicated by a red circle, centred on Takuanaupradoo, and by expanding the radius to include Koakoknongtungmae and Nongnamyen (p < 0.05); the 2^nd^, indicated by a yellow circle, centred on Mapchalood, and included Taladhauypong and Watsopol (p < 0.05); the 3^rd^, indicated by a magenta circle, centred on Koakoknongtungmae, and included Nongnamyen and Takuanaupradoo (p < 0.05).

**Figure 2:**
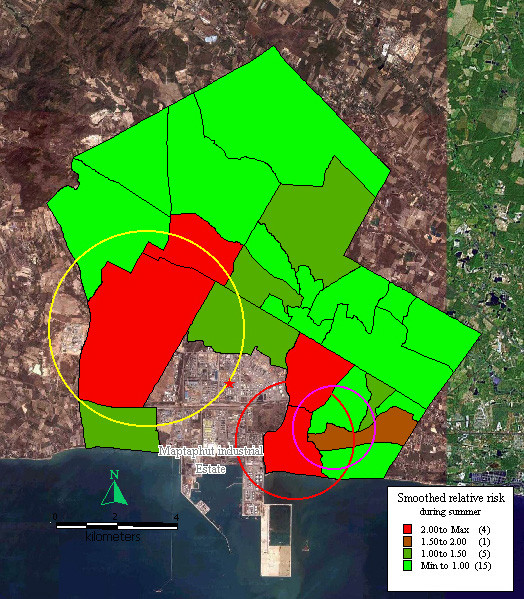
**Mapping of smoothed relative risk and disease clusters in the study area during summer**. Red circle indicates the first cluster of respiratory disease that includes Takuanaupradoo, Koakoknongtungmae and Nongnamyen. Yellow circle indicates the second cluster of respiratory disease that includes Mapchalood, Taladhauypong and Watsopol. Magenta circle indicates the third cluster of respiratory disease that includes Koakoknongtungmae, Nongnamyen and Takuanaupradoo. Red star sign indicates weighted mean centre of the stacks.

Focused cluster analysis found significant clusters near the stack weighted mean centre, indicated by a red star sign (p < 0.05), as shown in Figure 2.

During the rainy season, relative risk was > 2 in Takuanaupradoo (3.31), Taladhauypong (2.66), and Mapchalood (2.59). Relative risk was between 1.5–2 in Soiraumpattana (1.97) and Nongpab (1.84). Relative risk was between 1.0–1.5 in Baanplong (1.18), Klongnamhoo (1.18), Baanbon (1.17), Saklookya (1.11) and Islam (1.05). In the other communities, smoothed relative risk was < 1.0.

Disease formed three significant clusters, the 1^st^, indicated by a red circle, centred on Takuanaupradoo, and included Koakoknongtungmae and Nongnamyen (p < 0.05); the 2^nd^, indicated by a yellow circle, centred on Mapchalood, and included Taladhauypong and Watsopol (p < 0.05); the 3^rd^, indicated by a magenta circle, centred on Koakoknongtungmae, and included Nongnamyen and Takuanaupradoo (p < 0.05).

**Figure 3:**
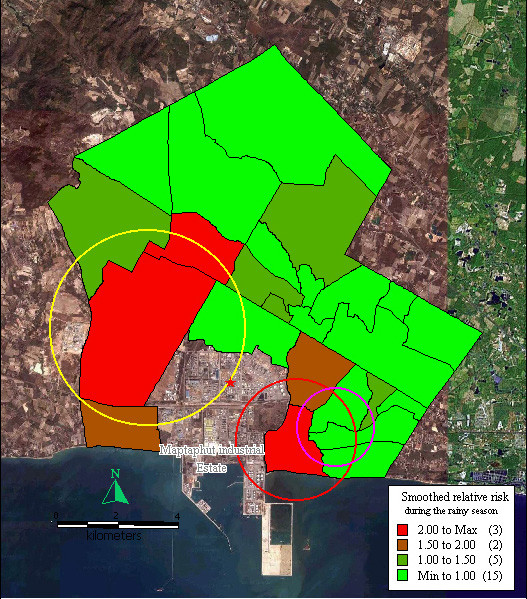
**Mapping of smoothed relative risk and disease clusters in the study area during the rainy season**. Red circle indicates the first cluster of respiratory disease that includes Takuanaupradoo, Koakoknongtungmae and Nongnamyen. Yellow circle indicates the second cluster of respiratory disease that includes Mapchalood, Taladhauypong and Watsopol. Magenta circle indicates the third cluster of respiratory disease that includes Koakoknongtungmae, Nongnamyen and Takuanaupradoo. Red star sign indicates weighted mean centre of the stacks.

Focused cluster analysis during the rainy season also found a significant cluster near the stack weighted mean centre, indicated by a red star sign (p < 0.05), as shown in Figure 3.

During winter, relative risk was > 2 in Takuanaupradoo (3.00), Mapchalood (2.98), Taladhauypong (2.58), and Nongpab (2.12). Relative risk was between 1.5–2 in Soiraumpattana (1.93), Islam (1.63), and Koakoknongtungmae (1.53). Relative risk was between 1.0–1.5 in Saklookya (1.15), Baanbon (1.10) and Baanplong (1.08). In the other communities, smoothed relative risk was < 1.5.

Disease formed three significant clusters, the 1^st^, indicated by a red circle, centred on Mapchalood, and included Taladhauypong and Watsopol (p < 0.05); the 2^nd^, indicated by a yellow circle, centred on Takuanaupradoo, and included Koakoknongtungmae and Nongnamyen (p < 0.05); the 3^rd^, indicated by a magenta circle, centred on Nongpab, and included Mapchalood, and Watsopol (p < 0.05).

**Figure 4:**
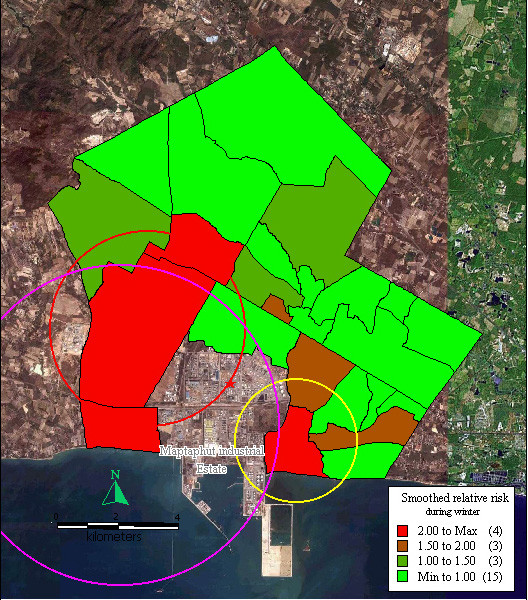
**Mapping of smoothed relative risk and disease clusters in the study area during winter**. Red circle indicates the first cluster of respiratory disease that includes Mapchalood, Taladhauypong and Watsopol. Yellow circle indicates the second cluster of respiratory disease and includes Takuanaupradoo, Koakoknongtungmea and Nongnamyen. Magenta circle indicates the third cluster of respiratory disease that includes Nongpab, Mapchalood and Watsopol. Red star sign indicates weighted mean centre of the stacks.

Focused cluster analysis during winter also found a significant cluster near the stack weighted mean centre, indicated by a red star sign (p < 0.05), as shown in Figure 4.

### Pollutant dispersion

**Figure 5:**
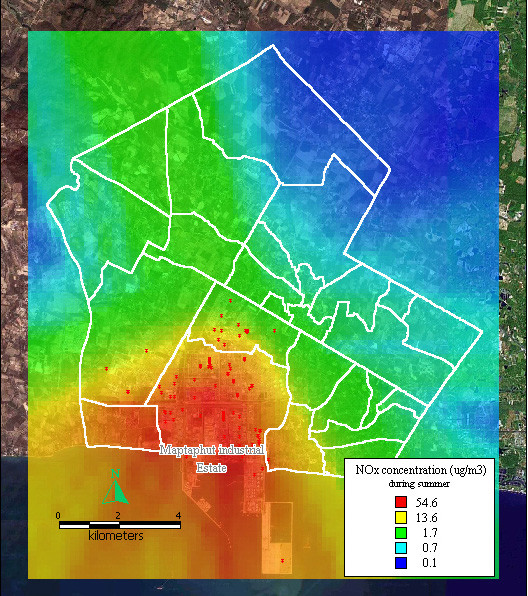
**Dispersion of NOx during summer**. Red dots indicate the stacks that released nitrogen oxide.

The dispersion of NO_x _during summer was highest in southwest Maptaphut Municipality, which included Takuanaupradoo, Mapchalood, and Nongpab, as shown in Figure 5.

**Figure 6:**
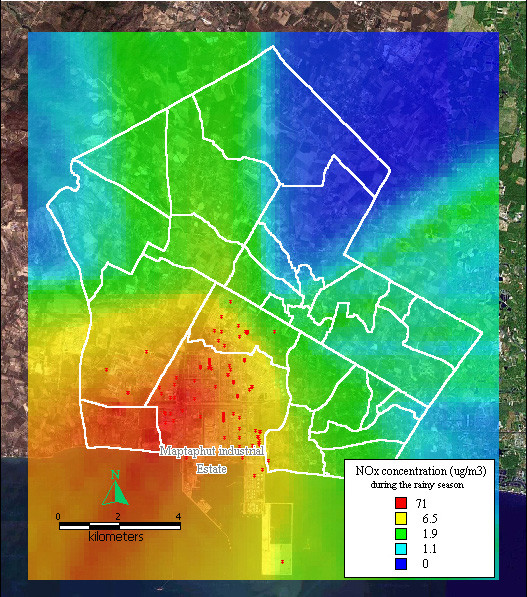
**Dispersion of NOx during rainy season**. Red dots indicate the stacks that released nitrogen oxide.

The dispersion of NO_x _during the rainy season was also highest in southwest Maptaphut Municipality, which includes Takuanaupradoo, Mapchalood, and Nongpab, as shown in Figure 6.

**Figure 7:**
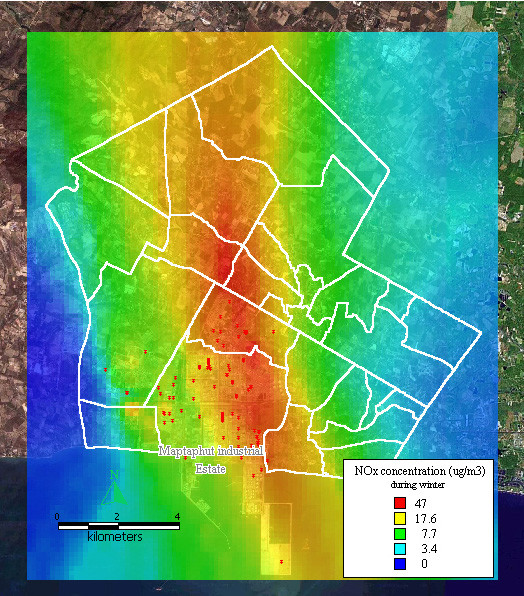
**Dispersion of NOx during winter**. Red dots indicate the stacks that released nitrogen oxide.

The dispersion of NO_x _during winter presented as a large band running through the middle of Maptaphut Municipality, including Takuanaupradoo, Mapchalood, Taladhauypong, Watsopol, Baanplong, and Hauypongnai, as shown in Figure 7.

**Figure 8:**
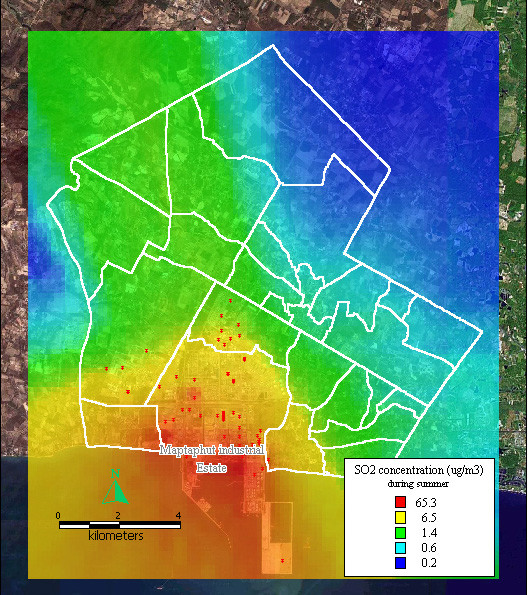
**Dispersion of SO2 during summer**. Red dots indicate the stacks that released sulfur dioxide.

**Figure 9:**
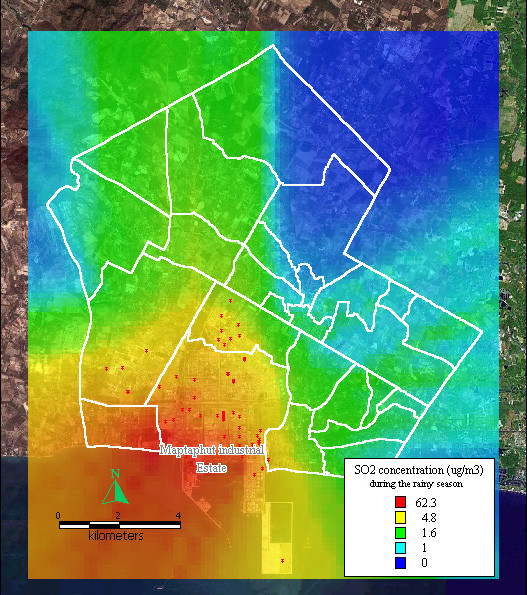
**Dispersion of SO2 during the rainy season**. Red dots indicate the stacks that released sulfur dioxide.

**Figure 10:**
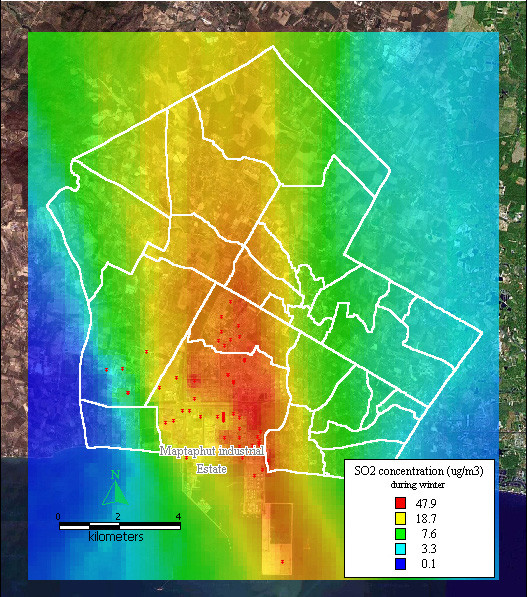
**Dispersion of SO2 during winter**. Red dots indicate the stacks that released sulfur dioxide.

**Figure 11:**
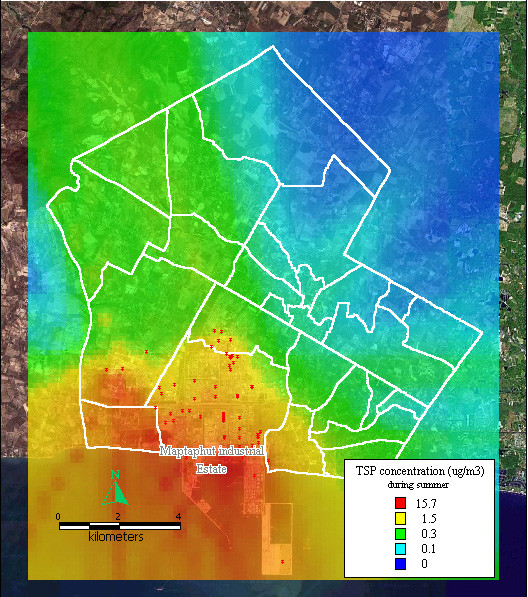
**Dispersion of TSP during summer**. Red dots indicate the stacks that released total suspended particle.

**Figure 12:**
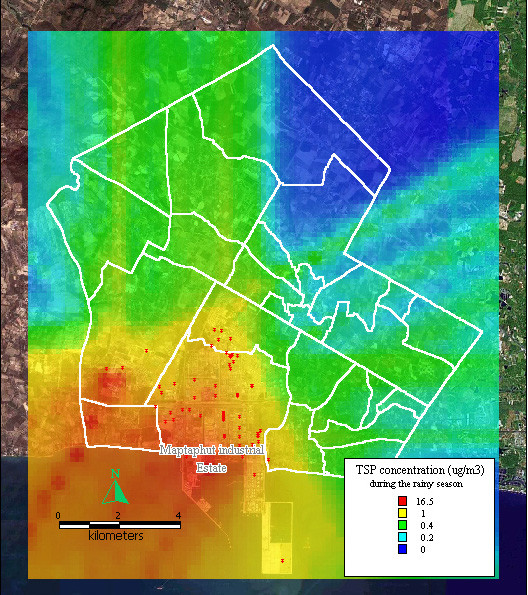
**Dispersion of TSP during the rainy season**. Red dots indicate the stacks that released total suspended particle.

**Figure 13:**
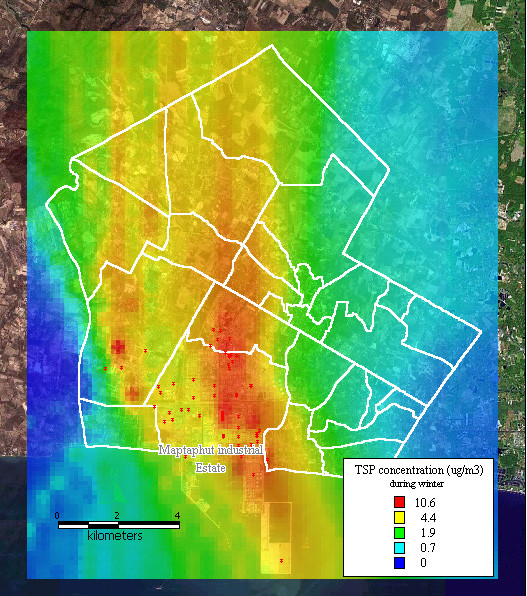
**Dispersion of TSP during winter**. Red dots indicate the stacks that released total suspended particle.

The dispersion of SO_2 _and TSP during summer, the rainy season, and winter, presented similar patterns to the dispersion of NO_x _during the same seasons, as shown in Figure 8, 9, 10, 11, 12, 13.

### Relationship between pollutants and disease occurrence

As shown in Table 2, pollutant concentrations were used to classify the communities into four groups. The rate of respiratory disease in the different community groups was recalculated and compared with the lowest concentration categories. During summer, the rainy season, and winter, the rates of disease and the pollutant concentrations showed a dose-response effect, where higher rates of disease occurred in communities with higher pollutant concentrations.

**Table 2:** Rate and relative risk in communities grouped by range of pollutants during summer, rainy season, and winter.

Summer					
Range of pollutants	Cases	population	Rate	RR	Communities

> 4.9	650	4633	140.30	5.64	22,23,13,9
2.3–4.9	307	4048	76.33	3.07	6,24,25,18,3
1.3–2.3	277	8380	33.15	1.33	19,2,5,8,20,14,17
< 1.3	214	8607	24.86	1.00	7,21,11,10,12,15,16,4,1

Rainy season					

Range of pollutants	Cases	population	Rate	RR	Communities

> 4.9	761	4633	164.26	5.15	22,23,13,9
2.3–4.9	527	8344	63.16	1.98	6,24,25,18,3,19,8,2
1.3–2.3	369	7019	52.57	1.65	10,15,5,14,17,10,7
< 1.3	181	5672	32.91	1.00	21,12,16,11,4,1

Winter					

Range of pollutants	Cases	population	Rate	RR	Communities

> 22.7	775	7312	105.99	2.67	13,8,6,2,23
12.0–22.7	400	4240	94.34	2.37	14,7,3,9
4.5 – 12.0	629	10343	61.81	1.53	1,11,5,17,24,18,4,19,25,10,20
< 4.5	150	3773	39.76	1.00	12,16,22,21,15

As illustrated in Table 3, the effects of total pollutant, NO_x_, SO_2 _and TSP were analysed by spatial regression analysis and by considering the distance from the communities to hospital and health office. The findings revealed that during summer total pollutant (p < 0.05), SO_2 _(p < 0.05) and NO_x _(p < 0.05) played a role in adverse health effects, after taking into account the distance from the communities to hospital and health office. During the rainy season, total pollutant (p < 0.05) and NO_x _(p < 0.05) played a role in adverse health effect, after taking into account distance. During winter, no relationship was found between pollutants and rate of respiratory disease, after taking into account distance. However, distances from communities to hospital and health office affected the reported rate of respiratory disease. It deceased the rate of respiratory disease during summer (p < 0.05), the rainy season (p < 0.05) and winter (p < 0.05).

**Table 3:** Spatial regression analysis between rates of respiratory disease and pollutant, and the distance from communities to hospital and health office.

Summer									
pollutant					pollutant and distance				

Variable	Coeff.	SE	z-value	p-value	Variable	Coeff.	SE	z-value	p-value

const.	0.0446	0.0080	5.6046	0.0000	const.	0.1045	0.0245	4.2664	0.0000
Pol	0.0032	0.0010	3.2337	0.0012	Pol	0.0028	0.0009	3.1426	0.0017
					avedis	-0.0126	0.0049	-2.5463	0.0109
const.	0.0534	0.0113	4.7701	0.0000	const.	0.1284	0.0310	4.1427	0.0000
TSP	0.0038	0.0051	0.7398	0.4594	TSP	0.0046	0.0045	1.0106	0.3122
					avedis	-0.0159	0.0063	-2.5507	0.0108
const.	0.0409	0.0076	5.4068	0.0000	const.	0.0971	0.0232	4.1964	0.0000
SO2	0.0125	0.0032	3.9006	0.0001	SO2	0.0109	0.0029	3.7228	0.0002
					avedis	-0.0117	0.0046	-2.5233	0.0116
const.	0.0404	0.0068	5.9647	0.0000	const.	0.0915	0.0211	4.3448	0.0000
NOx	0.0084	0.0018	4.6049	0.0000	NOx	0.0076	0.0017	4.5734	0.0000
					avedis	-0.0107	0.0042	-2.5279	0.0115

Rainy season									

pollutant					pollutant and distance				

Variable	Coeff.	SE	z-value	p-value	Variable	Coeff.	SE	z-value	p-value

const.	0.0581	0.0120	4.8358	0.0000	const.	0.1342	0.0341	3.9329	0.0001
Pol	0.0026	0.0011	2.1685	0.0301	Pol	0.0025	0.0011	2.2962	0.0217
					avedis	-0.0160	0.0068	-2.3513	0.0187
const.	0.0635	0.0144	4.4069	0.0000	const.	0.1464	0.0378	3.8778	0.0001
TSP	0.0122	0.0122	1.0007	0.3170	TSP	0.0150	0.0111	1.3519	0.1764
					avedis	-0.0177	0.0076	-2.3329	0.0197
const.	0.0624	0.0138	4.5339	0.0000	const.	0.1435	0.0374	3.8353	0.0001
SO2	0.0036	0.0028	1.2863	0.1984	SO2	0.0037	0.0025	1.4865	0.1371
					avedis	-0.0172	0.0074	-2.2975	0.0216
const.	0.0521	0.0099	5.2656	0.0000	const.	0.1187	0.0292	4.0626	0.0001
NOx	0.0082	0.0023	3.5237	0.0004	NOx	0.0076	0.0021	3.5958	0.0003
					avedis	-0.0139	0.0059	-2.3935	0.0167

Winter									

pollutant					pollutant and distance				

Variable	Coeff.	SE	z-value	p-value	Variable	Coeff.	SE	z-value	p-value

const.	0.0451	0.0250	1.8020	0.0716	const.	0.1572	0.0633	2.4825	0.0131
Pol	0.0021	0.0014	1.4637	0.1433	Pol	0.0008	0.0015	0.4878	0.6257
					avedis	-0.0197	0.0103	-1.9164	0.0553
const.	0.0516	0.0250	2.0621	0.0392	const.	0.1611	0.0552	2.9172	0.0035
TSP	0.0158	0.0142	1.1112	0.2665	TSP	0.0074	0.0138	0.5380	0.5906
					avedis	-0.0205	0.0093	-2.1921	0.0284
const.	0.0390	0.0255	1.5301	0.1260	const.	0.1476	0.0688	2.1450	0.0320
SO2	0.0058	0.0033	1.7412	0.0817	SO2	0.0023	0.0038	0.6082	0.5430
					avedis	-0.0184	0.0109	-1.6955	0.0900
const.	0.0510	0.0242	2.1090	0.0350	const.	0.1646	0.0599	2.7473	0.0060
NOx	0.0037	0.0030	1.2334	0.2174	NOx	0.0011	0.0030	0.3609	0.7182
					avedis	-0.0205	0.0099	-2.0542	0.0399

const. = constant, coeff = coefficient, SE, = standard error, Pol = total pollutant, TSP = total suspended particle, SO2 = sulfur dioxide, NOx = nitrogen oxide, avedis = average distance from community to hospital and health office

As shown in Table 4, six communities–Mapchalood, Taladhauypong, Watsopol, Takuanaupradoo, Koakoknongtungmae, and Nongpab–were selected based on clustering and distribution of pollutants during the three seasons. A time series analysis was performed on the selected communities for a one-year period. Previous-day concentrations of total pollutants, after taking into account previous day volume of rainfall, played a non-significant role on rate of respiratory disease per 1,000 people (p = 0.051). However, after taking into account previous day volume of rainfall, same-day concentrations of SO_2 _affected the rate; a change of 1 microgram per cubic meter changed the rate by 0.0123 (p = 0.0285). Previous-day concentrations of NO_x _affected the rate of respiratory disease per 1,000 people; a change of 1 microgram per cubic meter changed the rate by 0.0124 (p = 0.0379). In addition, previous-day concentration of TSP affected the rate of respiratory disease per 1,000 people; a change of 1 microgram per cubic meter would change the rate by 0.0398 (p = 0.0262). Rainfall also had a significant effect on the rate of respiratory disease since it decreased the rate about 0.008 per 1,000 people with every 1 millimetres of previous-day rainfall.

**Table 4:** Time series analysis between rate of respiratory disease and pollutant.

Variables	Coefficient	Std. Error	T-statistics	p-value
AR	0.1052	0.0527	1.9979	0.0465
Pollutant_(t-1)_	0.0054	0.0028	1.9621	0.0505
Rain_(t-1)_	-0.0081	0.0029	-2.8477	0.0047
Constant	1.2382	0.0586	21.1162	0.0000
				

Variables	Coefficient	Std. Error	T-statistics	p-value

AR	0.1113	0.0526	2.1163	0.0350
TSP_(t-1)_	0.0398	0.0178	2.2307	0.0262
Rain_(t-1)_	-0.0082	0.0028	-2.8861	0.0041
Constant	1.2523	0.0509	24.5845	0.0000
				

Variables	Coefficient	Std. Error	T-statistics	p-value

AR	0.0994	0.0528	1.8825	0.0606
SO_2__(t)_	0.0123	0.0056	2.1986	0.0285
Rain_(t-1)_	-0.0079	0.0028	-2.7681	0.0059
Constant	1.2468	0.0526	23.7134	0.0000
				

Variables	Coefficient	Std. Error	T-statistics	p-value

AR	0.1094	0.0525	2.0829	0.0380
N0_x(t-1)_	0.0124	0.006	2.0837	0.0379
Rain_(t-1)_	-0.008	0.0028	-2.83	0.0049
Constant	1.2272	0.0607	20.2065	0.0000

AR = auto-correlation term, pollutant_(t-1) _= total pollutant concentration on previous day, rain_(t-1) _= rainfall at previous day, TSP _(t-1) _= total suspended particle at previous day, SO_2__(t) _= sulfur dioxide at the same day, NO_x(t-1) _= nitrogen oxide at previous day.

## Discussion

The rate of disease was smoothed to correct the stability of any extreme values that might occur in an area with a small population. The relative risk presented the excess risk in the communities, and aimed to determine areas at risk.

The rate of respiratory disease was lower in summer (56.49/1,000), higher during the rainy season (71.61/1,000), and highest during winter (76.13/1,000). A possible explanation is that during winter, it was colder, and that caused people to contract respiratory diseases directly, and/or the cold weather reduced pollutant dispersion exposing the population to higher concentrations of pollutants in their areas resulting in increased respiratory disease.

Disease clustering was done by using a scan technique, a circular window centred on each community in turn and expanded to include neighbouring regions until the total aggregated population within the window equals a user-defined threshold. In this study, during summer, the circular window was centred at high-rate areas, such as Takuanaupradoo (rate = 190.72) and expanded to include neighbouring regions such as Koakoknongtungmae (rate = 91.01) and Nongnamyen (rate = 5.63) to form the cluster. Nongnamyen was included in the cluster even though the rate of respiratory disease was low. During summer, clusters were located at Takuanaupradoo, Mapchalood, and Koakoknongtungmae. During the rainy season, the clusters were similar to summer, at Takuanaupradoo, Mapchalood and Koakoknongtungmae. During winter, clusters were located at Mapchalood, Takuanaupradoo, and Nongpab.

Disease mapping represented the excess risk of respiratory disease in areas near Maptaphut Industrial Estate during the three seasons. The finding was supported by disease cluster analysis, which found disease formed significant clusters in communities close to the estate (p < 0.05). In addition, the disease clusters were related to the weighted mean centre of the chimney stacks that released pollutants into the communities (p < 0.05).

Pollutant dispersions were based on both wind speed and wind direction. During summer and the rainy season, pollutants mostly dispersed out to the sea while during winter, pollutants blew and dispersed towards the communities exposing the population to higher concentrations of pollutants than other seasons. Thus rate of respiratory disease was highest during winter.

Spatial analysis was used to determine the relationship of pollutant concentration and rate of respiratory disease in the same community by taking the distance between community and health providers into account. The result revealed the effect of distance on decreasing the rate of respiratory disease during summer, the rainy season, and winter. During summer, total pollutant (p < 0.05), SO_2 _(p < 0.05) and NO_x _(p < 0.05) played a role in adverse health effect after taking into account distance. During the rainy season, total pollutant (p < 0.05) and NO_x _(p < 0.05) played a role in adverse health effects, after taking into account distance. However, during winter, no relationship was found between pollutant and rate of respiratory disease, after taking into account distance.

To minimize potential bias, the six communities included in the disease clusters which received the greatest impact from pollutant dispersion, were selected for time-series analysis. The finding revealed that, after taking into account rainfall, the rate of respiratory disease was influenced by the concentration of SO_2 _on the same day, or NO_x _or TSP on the previous day.

## Conclusion

This paper has suggested a technique for analysing spatial data for respiratory disease, and related the rate of respiratory disease to estimated pollutants.

The distribution of disease presents a high excess risk in communities near Maptaphut Industrial Estate. Disease clusters also exist adjacent to the estate and are related to the weighted mean centre of the chimney stacks. The relationship between estimated pollutants and rate of respiratory disease was shown to be significant by using a time-series analysis of the six communities selected by disease clustering, and subject to the greatest impact from pollutant dispersion.

The effects of the pollutants maybe summarized as follows: 1 microgram per cubic meter of SO_2 _on the current day could change the rate of respiratory disease per 1,000 people by 0.0123; 1 microgram per cubic meter of NO_x _on the previous day could change the rate by 0.0124; 1 microgram per cubic meter of TSP on the previous day could change the rate by 0.0398.

## Methods

### Study area and data used

**Figure 14:**
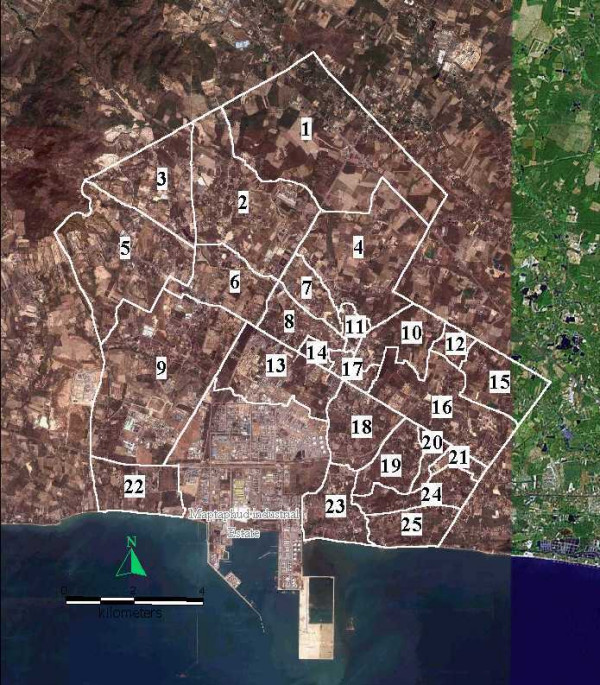
**The Maptaphut Municipality composes of 25 communities**. White polygons indicate community boundaries. 1 = Mapka 2 = Hauypongnai 3 = Nongwaisom 4 = Baanbon 5 = Saklookya 6 = Taladhauypong 7 = Mapya 8 = Baanplong 9 = Mapchalood 10 = Baanlang 11= Watmaptaphut 12 = Samnukkabak 13 = Watsopol 14 = Islam 15 = Kaopai 16 = Kodehin 17 = Taladmaptaphut 18 = Soiraumpattana 19 = Nongnamyen 20 = Klongnamhoo 21 = Nongbaudang 22 = Nongpab 23 = Takuanaupradoo 24 = Koakoknongtungmae 25 = Krokyaicha

Maptaphut Municipality, located in Rayong Province, consists of 25 communities (see Figure 14); the area surrounds the Maptaphut Industrial Estate, where about 228 chimney stacks are located. The main pollutants released by the stacks are sulfur dioxide (SO_2_), nitrogen oxides (NO_x_), and total suspended particles (TSP).

### Medical data

Medical data were obtained from Maptaphut Hospital and Maptaphut Health Office, both located in the study area. Cases of respiratory disease were defined as persons who had resided in Maptaphut Municipality and had presented with any symptom of respiratory disease listed in Chapter 10 of ICD-10 [8]. Medical data from the hospital was coded by medical recording officers, while data from the health office were coded by a well-trained nurse. The case count was recorded daily from 1^st ^March 2000 to 28^th ^February 2001. The medical data was divided into 3 periods–l^st ^March 2000-30^th ^June 2000 (summer), 1^st ^July 2000-31^st ^October 2000 (rainy season), and 1^st ^November 2000-28^th ^February 2001 (winter).

### Pollutant source data

The data set was obtained from a report of the study of mathematical modelling in assessing air pollutant problems that impact health in Maptaphut Industrial Estate and surrounding area, which was submitted to the Department of Health, Ministry of Public Health, in 2002. The data included chimney stack locations in Universal Transverse Mercator (UTM) coordinates, level of stack base from average sea level, stack characteristics, i.e., height (meter), diameter (meter), releasing temperature at stack tip (Kelvin), gas velocity at stack tip (meter per second), and amount of pollutants, i.e., TSP, SO_2_, NO_x _released from stack tip (gram per second).

### Meteorological data

Meteorological data recorded in 2000 and 2001 were obtained from the Department of Meteorology, Bangna, Bangkok. The data included daily rainfall and temperature recorded at the Meteorological Station located in Hauypong community, in the middle of the study area. The data also included wind speed, wind direction and cloud data (Deca) recorded every 3 hours at Rayong Meteorological Centre.

### Pollutant data

Estimated pollutant data, obtained by air modelling, included sulfur dioxide (SO_2_), nitrogen oxide (NO_x_) and total suspended particles (TSP). Two types of pollutant data were estimated as follows.

- Pollutant concentrations were estimated at 500 metre intervals that included 1,184 points of estimation to cover the study area. The estimates were calculated three times to represent pollutant dispersion during summer, the rainy season and winter. Each point of concentration was used for interpolating and presenting continuous pollutant dispersions using grid analysis by GIS software.

- Daily pollutant concentrations were estimated at the centroid of communities from 1^st ^March 2000 to 28^th ^February 2001. The data was used to determine the relationship between pollutants and disease occurrence.

## Methodology

### Smoothed rate and smoothed relative risk

Smoothed rate and relative risk was estimated by Bayesian statistics [9-13] by running the following model in WinBUGS [14].

O_[i] _~ dpois(mu_[i]_)

log(mu_[i]_) <- log(E_[i]_) + α_0 _+ α_1_·X_1[i] _+ α_2_·X_2[i] _+ b_[i]_

Rate in area i = mu_[i]_/population in area i × 1,000

Relative risk in area i = mu_[i]_/E_[i]_

Where; O_[i] _= observed case count in area i

E_[i] _= expected case count in area i

= (summation of cases × population in area i)/Total population

X_1[i] _= Pollutant concentration in area i.

X_2[i] _= Average distance from hospital and health office to area i.

b_[i] _= Spatial effect of neighbouring locations of area i

### Disease mapping and disease clustering

Global clustering: Besag and Newell's method [15-18].

Local clustering: Turnbull's Method [19]; and constant population size of each circular window was assigned as 2,500 people.

Focused clustering: Score Test of Lawson and Waller [20]; the putative source was estimated from the weighted mean centre of 228 stacks (East 733,469 m North 1,404,378 m).

### Pollutant estimation

Pollutant concentrations (NOx, SO_2 _and TSP) were estimated using a Gaussian plume model, by ISCST_3 _[21,22] software recommended by USEPA [23]. The model used to estimate pollutant concentration is shown as follows.

c=q2πuσzσyexp⁡(−y22σy2)[exp⁡(−(z−h)22σz2)−exp⁡(−(z+h)22σz2)]
 MathType@MTEF@5@5@+=feaafiart1ev1aaatCvAUfKttLearuWrP9MDH5MBPbIqV92AaeXatLxBI9gBaebbnrfifHhDYfgasaacH8akY=wiFfYdH8Gipec8Eeeu0xXdbba9frFj0=OqFfea0dXdd9vqai=hGuQ8kuc9pgc9s8qqaq=dirpe0xb9q8qiLsFr0=vr0=vr0dc8meaabaqaciaacaGaaeqabaqabeGadaaakeaacqWGJbWycqGH9aqpdaWcaaqaaiabdghaXbqaaiabikdaYGGaciab=b8aWjabdwha1jab=n8aZnaaBaaaleaacqWG6bGEaeqaaOGae83Wdm3aaSbaaSqaaiabdMha5bqabaaaaOGagiyzauMaeiiEaGNaeiiCaa3aaeWaaeaacqGHsisldaWcaaqaaiabdMha5naaCaaaleqabaGaeGOmaidaaaGcbaGaeGOmaiJae83Wdm3aa0baaSqaaiabdMha5bqaaiabikdaYaaaaaaakiaawIcacaGLPaaadaWadaqaaiGbcwgaLjabcIha4jabcchaWnaabmaabaGaeyOeI0YaaSaaaeaacqGGOaakcqWG6bGEcqGHsislcqWGObaAcqGGPaqkdaahaaWcbeqaaiabikdaYaaaaOqaaiabikdaYiab=n8aZnaaDaaaleaacqWG6bGEaeaacqaIYaGmaaaaaaGccaGLOaGaayzkaaGaeyOeI0IagiyzauMaeiiEaGNaeiiCaa3aaeWaaeaacqGHsisldaWcaaqaaiabcIcaOiabdQha6jabgUcaRiabdIgaOjabcMcaPmaaCaaaleqabaGaeGOmaidaaaGcbaGaeGOmaiJae83Wdm3aa0baaSqaaiabdQha6bqaaiabikdaYaaaaaaakiaawIcacaGLPaaaaiaawUfacaGLDbaaaaa@7267@

Where, c = Chemical concentrations at the specified location (g/m^3^)

q = Rate of chemical emission from stack (g/s)

u = Wind speed at stack terminal (m/s)

σ_y _= Standard deviation in y direction or lateral dispersion coefficient (m)

σ_z _= Standard deviation in z direction or vertical dispersion coefficient (m)

y = Distance along a horizontal axis perpendicular to the wind

z = Distance along a vertical axis

h = Effective stack height (physical stack height plus plume rise)

### Relationship of respiratory disease occurrence and estimated pollutants using spatial regression analysis

Spatial regression analysis was analyzed by GeoDA to determine the relationship between the estimated pollutants that impact the area, and the rate of respiratory disease. The model used for this analysis is as follows:

Y_[i] _= α_0 _+ α_1_·X_1[i] _+ α_2_·X_2[i]_+ b_[i]_

Where Y_[i] _is the empirical Bayes smoothed rate of respiratory disease in area i. X_1[i] _is the pollutant that impacts area i. X_2[i] _is the average distance from hospital and health office to area i. The α_1 _and α_2 _are the regression coefficients that represent the effect of X_1 _and X_2 _on Y respectively. b_[i] _is the term for spatial autocorrelation and the assumption is made that the rates of respiratory disease in the areas were correlated to each other. The autocorrelation was then adjusted by spatial error in the regression analysis and spatial weight was constructed as Queen types of contiguity.

### Relationship of respiratory disease occurrence and estimated pollutants using time-series analysis

A time series was employed to determine the relationship between estimated pollutants and rate of respiratory disease over time, taking autocorrelation into account. The model for this is expressed as follows:

Y_[t] _= α_0 _+ α_1_·X_1[t-m] _+ α_2_·X_2[t-n]_

Y_[t] _is the rate of respiratory disease at time t. α_1 _and α_2 _are regression coefficient represent the effect of X_1 _and X_2_·X_1 _and X_2 _are independent variables such as pollutant and rainfall. The m and n are the lag time of variable X_1 _and X_2 _respectively.

## Conflict of interest

The author(s) declare that they have no competing interests.

## Authors' contributions

Authors SJ, PS, JK, RS, SS and SK collaborated intensely on all aspects of the manuscript-research design, data preparation, statistical analysis, and discussion.
